# Supplementary material for: ZFP148 is a transcriptional repressor of cytolytic effector CD8+ T cell differentiation
Source: Nat Immunol. 2026 Mar 27;27(4):827–40. doi: 10.1038/s41590-026-02461-2 (PMC13043298; doi:10.1038/s41590-026-02461-2)
Supplement: Supplementary file 1 — Supplementary Fig. 1 gating strategy. [file 41590_2026_2461_MOESM1_ESM.pdf]

nature immunology

Supplementary information https://doi.org/10.1038/s41590-026-02461-2

ZFP148 is a transcriptional repressor of cytolytic effector CD8$^+$ T cell differentiation

In the format provided by the authors and unedited

a

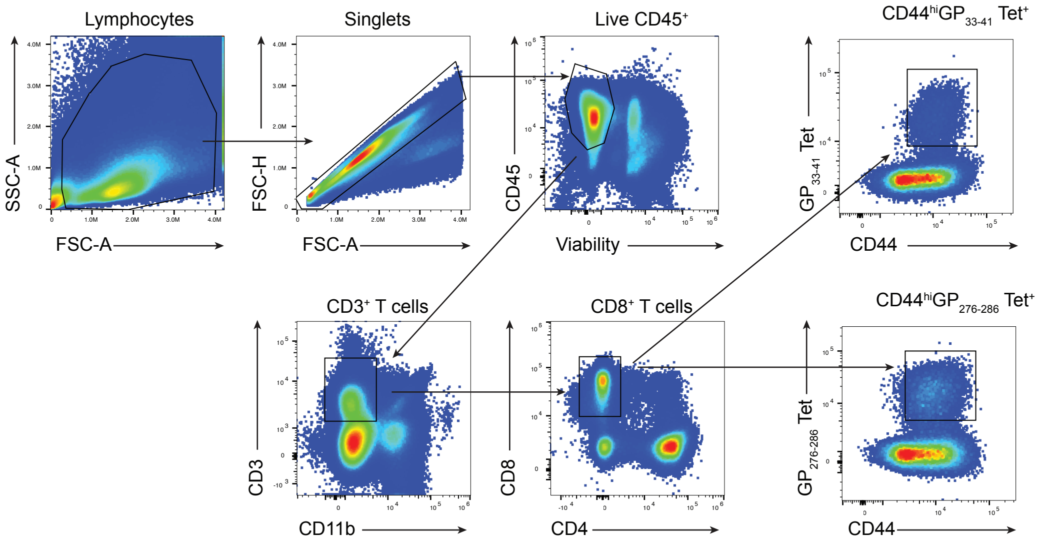

Gating strategy for analyzing CD44^{hi}GP$_{33-41}$ Tet^{+} or CD44^{hi}GP$_{276-286}$ Tet^{+} CD8^{+} T cells in spleens or inguinal lymph nodes of C57BL/6 WT mice or *Zfp148*$^{fl/fl}$ mice or ZFP148 cKO mice post-LCMV Cl13 or LCMV Armstrong infection

b

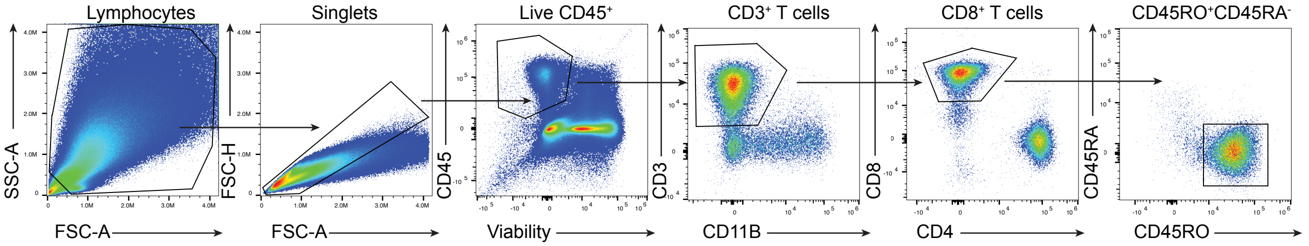

Gating strategy for analyzing CD45RO^{+}CD45RA^{+} activated tumor-infiltrating CD8^{+} T cells in human muscle invasive bladder cancer specimens

c

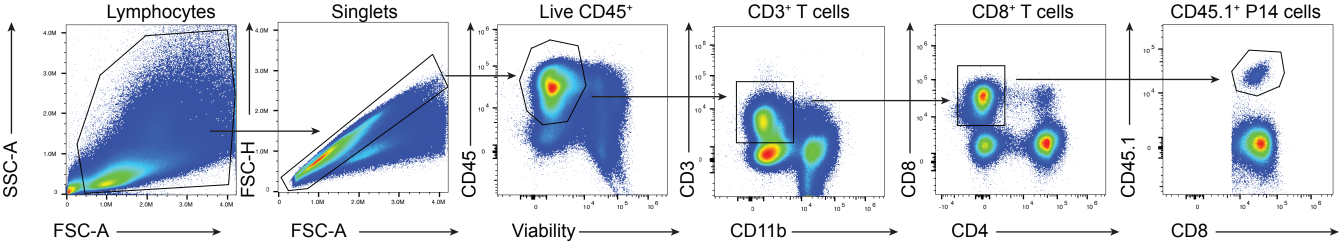

Gating strategy for analyzing transferred P14 or P14 KLF2-EGFP CD8^{+} T cells in spleens of C57BL/6 WT recipient mice at day 21-post T cell transfer and LCMV Cl13 infection

d

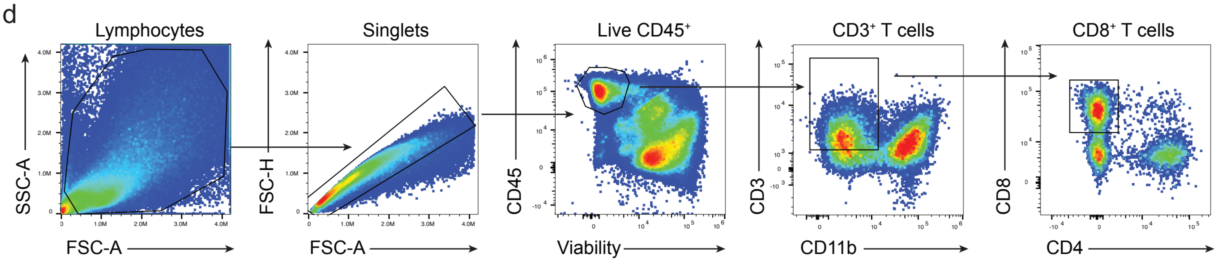

Gating strategy for analyzing tumor-infiltrating CD8^{+} T cells in syngeneic mouse colon adenocarcinoma MC38 tumors
