# Supplementary material for: ZFP148 is a transcriptional repressor of cytolytic effector CD8+ T cell differentiation
Source: Nat Immunol. 2026 Mar 27;27(4):827–40. doi: 10.1038/s41590-026-02461-2 (PMC13043298; doi:10.1038/s41590-026-02461-2)
Supplement: Supplementary file 2 — Reporting Summary [file 41590_2026_2461_MOESM2_ESM.pdf]

nature portfolio

Corresponding author(s): Zihai Li

Last updated by author(s): Feb 10, 2026

Reporting Summary

Nature Portfolio wishes to improve the reproducibility of the work that we publish. This form provides structure for consistency and transparency in reporting. For further information on Nature Portfolio policies, see our Editorial Policies and the Editorial Policy Checklist.

Statistics

For all statistical analyses, confirm that the following items are present in the figure legend, table legend, main text, or Methods section.

n/a	Confirmed	
☐	☒	The exact sample size (*n*) for each experimental group/condition, given as a discrete number and unit of measurement
☐	☒	A statement on whether measurements were taken from distinct samples or whether the same sample was measured repeatedly
☐	☒	The statistical test(s) used AND whether they are one- or two-sided<br>*Only common tests should be described solely by name; describe more complex techniques in the Methods section.*
☐	☒	A description of all covariates tested
☐	☒	A description of any assumptions or corrections, such as tests of normality and adjustment for multiple comparisons
☐	☒	A full description of the statistical parameters including central tendency (e.g. means) or other basic estimates (e.g. regression coefficient) AND variation (e.g. standard deviation) or associated estimates of uncertainty (e.g. confidence intervals)
☐	☒	For null hypothesis testing, the test statistic (e.g. *F*, *t*, *r*) with confidence intervals, effect sizes, degrees of freedom and *P* value noted<br>*Give P values as exact values whenever suitable.*
☒	☐	For Bayesian analysis, information on the choice of priors and Markov chain Monte Carlo settings
☒	☐	For hierarchical and complex designs, identification of the appropriate level for tests and full reporting of outcomes
☐	☒	Estimates of effect sizes (e.g. Cohen's *d*, Pearson's *r*), indicating how they were calculated

Our web collection on statistics for biologists contains articles on many of the points above.

Software and code

Policy information about availability of computer code

Data collection	Flow cytometry: Cytek® Aurora<br>Cell sorting: Cytek Aurora™ CS System<br>Matched scRNA-seq and scATAC-seq: The illumina Novaseq X plus 840 platform<br>CUT&Tag-seq: The illumina Novaseq X plus 840 platform
Data analysis	Statistical analyses for flow cytometry, tumor growth curves, and mouse survival were performed using GraphPad Prism (v.10). Unpaired or paired two-sided t-tests were used for comparisons between two unpaired or paired groups, respectively. One-way ANOVA followed by Tukey's multiple-comparisons test was used for comparisons among three or more groups. One-way ANOVA followed by Holm–Šídák's multiple-comparisons test was used for comparisons between pre-selected pairs among three or more groups. Two-way ANOVA was used to compare time-course curves, with Bonferroni correction for multiple comparisons. The log-rank test was used to compare overall survival of mice across multiple treatment groups, with Bonferroni correction for multiple comparisons.<br>Analyses of mouse scRNA-seq and scATAC-seq data were performed using R (v.4.4.0) with the packages Seurat (v.5.1.0), Signac (v.1.14.0), AUCell (v.1.26.0), slingshot (v.2.12.0), chromVAR (v.1.26.0), clusterProfiler (v.4.12.6), ComplexHeatmap (v.2.20.0), and EnhancedVolcano (v.1.22.0). Differentially expressed genes were identified using the FindMarkers() or FindAllMarkers() functions in Seurat, with statistical significance assessed by a two-sided Wilcoxon rank-sum test, with Benjamini–Hochberg correction for multiple comparisons. Differentially accessible chromatin regions (DACRs) and transcription factor motif accessibility were identified using FindMarkers() or FindAllMarkers() with statistical significance assessed by a two-sided logistic regression likelihood-ratio test, with Benjamini–Hochberg correction for multiple comparisons. Gene Ontology enrichment analysis was performed using a one-sided hypergeometric test, with Benjamini–Hochberg correction for multiple comparisons. A two-sided Wilcoxon rank-sum test was used to compare mRNA expression, promoter chromatin accessibility, motif accessibility, and gene signature scores between Zfp148fl/fl and ZFP148 cKO CD8⁺ T cells. Analyses of the integrated human scRNA-seq

dataset were performed using Python (v.3.10.9) packages Scanpy (v.1.9.5), Pandas (v.2.0.0), Statsmodels (v.0.14.0), NumPy (v.1.24.2), SciPy (v.1.10.1), Matplotlib (v.3.8.0), Seaborn (v.0.11.2), and scikit-learn (v.1.3.2), as well as R (v.4.3.1) packages Circlize (v.0.4.16), GseaVis (v.0.0.5), Enrichplot (v.1.22.0), GridExtra (v.2.3.0), pheatmap (v.1.0.12), and DEGreport (v.1.38.5). A two-sided Wilcoxon rank-sum test was used for comparisons between two groups. Overall survival between two groups of patients was compared using the log-rank test. A P value ≤ 0.05 (or adjusted P value ≤ 0.05 after multiple-testing correction) was considered statistically significant.

For manuscripts utilizing custom algorithms or software that are central to the research but not yet described in published literature, software must be made available to editors and reviewers. We strongly encourage code deposition in a community repository (e.g. GitHub). See the Nature Portfolio guidelines for submitting code & software for further information.

Data

Policy information about availability of data

All manuscripts must include a data availability statement. This statement should provide the following information, where applicable:

- Accession codes, unique identifiers, or web links for publicly available datasets
- A description of any restrictions on data availability
- For clinical datasets or third party data, please ensure that the statement adheres to our policy

Parallel scRNA-seq and scATAC-seq and CUT&Tag-seq data are available in the NCBI database under accession numbers GSE297040 and GSE296311, respectively. Source data are provided with this paper. Further information and requests for data should be directed to the corresponding author, Z. Li.

Research involving human participants, their data, or biological material

Policy information about studies with human participants or human data. See also policy information about sex, gender (identity/presentation), and sexual orientation and race, ethnicity and racism.

Reporting on sex and gender	N/A All blood and tumor samples were completely deidentified.
Reporting on race, ethnicity, or other socially relevant groupings	N/A All blood and tumor samples were completely deidentified.
Population characteristics	The patient cohort had a median age of 68 years.
Recruitment	The consenting treatment-naive patient was prospectively enrolled in The Ohio State University Comprehensive Cancer Center bladder cancer tissue registry (Buck-IRB 2018C0181).
Ethics oversight	The Ohio State University Comprehensive Cancer Center's bladder cancer tissue registry (Buck-IRB 2018C0181).

Note that full information on the approval of the study protocol must also be provided in the manuscript.

Field-specific reporting

Please select the one below that is the best fit for your research. If you are not sure, read the appropriate sections before making your selection.

☒ Life sciences ☐ Behavioural & social sciences ☐ Ecological, evolutionary & environmental sciences

For a reference copy of the document with all sections, see nature.com/documents/nr-reporting-summary-flat.pdf

Life sciences study design

All studies must disclose on these points even when the disclosure is negative.

Sample size	No statistical methods were used to predetermine sample size. Sample sizes are indicated in the figure legends and were similar to those reported in previous publications, as referenced in the Methods section.
Data exclusions	No data were excluded from this study.
Replication	All data were generated from at least two independent experiments with a minimum of three biological replicates, yielding consistent phenotypes to ensure reproducibility, with the exception of the parallel scRNA-seq and scATAC-seq on CD44hiGP33-41 Tet+CD8+ T cells in spleens of Zfp148fl/fl and ZFP148 cKO mice at day 21 post-LCMV Cl13 infection. To minimize inter-mouse variability and enhance reproducibility, cells used for the single-cell multiome experiment were pooled from 10 mice for Zfp148fl/fl and 12 mice for ZFP148 cKO.
Randomization	Age- and sex-matched animals were randomly assigned to experimental conditions.
Blinding	Blinding was not performed due to the necessity of cage labeling and the use of mice with distinct genotypes and/or sexes. Investigators were

Blinding	not blinded to group allocation in order to maintain clarity during treatment administration and other experimental procedures.

Reporting for specific materials, systems and methods

We require information from authors about some types of materials, experimental systems and methods used in many studies. Here, indicate whether each material, system or method listed is relevant to your study. If you are not sure if a list item applies to your research, read the appropriate section before selecting a response.

Materials & experimental systems

n/a	Involved in the study
☐	☒ Antibodies
☐	☒ Eukaryotic cell lines
☒	☐ Palaeontology and archaeology
☐	☒ Animals and other organisms
☒	☐ Clinical data
☒	☐ Dual use research of concern
☒	☐ Plants

Methods

n/a	Involved in the study
☐	☒ ChIP-seq
☐	☒ Flow cytometry
☒	☐ MRI-based neuroimaging

Antibodies

Antibodies used

Flow cytometry antibodies
Mouse CD8 BUV496 53-6.7 1:400 BD 569181
Mouse CD3 BUV737 145-2C11 1:200 BD 612771
Mouse CD45 BV510 30-F11 1:100 BioLegend 103138
Mouse CD25 BB515 PC61 1:200 BD 564424
Mouse PD-1 FITC J43 1:200 eBioscience 11-9985-85
Mouse PD-1 Percp-Fire 806 29F.1A12 1:200 BioLegend 135262
Mouse CD11b Alexa Fluor 532 M1/70 1:800 eBioscience 58-0112-82
Mouse Ki-67 BUV395 B56 1:200 BD 564071
Mouse CD27 BUV563 LG.3A10 1:400 BD 741275
Mouse GITR BUV615 DTA-1 1:800 BD 751532
Mouse CD44 BUV661 IM7 1:400 BD 741471
Mouse LAG-3 BUV805 C9B7W 1:100 BD 748540
Mouse CD62L BV421 MEL-14 1:400 BioLegend 104436
Mouse ICOS Super Bright 436 C398.4A 1:400 eBioscience 62-9949-82
Mouse CD95 BV480 Jo2 1:200 BD 746755
Mouse KLRG1 Pacific Orange 2F1 1:200 eBioscience 79-5893-82
Mouse KLRG1 BV605 2F1/KLRG1 1:200 BioLegend 138419
Mouse VISTA Super Bright 600 MIH64 1:400 eBioscience 63-1083-82
Mouse TIGIT BV650 1G9 1:200 BD 744213
Mouse TIM-3 BV711 RMT3-23 1:200 BioLegend 119727
Mouse CD38 BV750 90/CD38 1:400 BD 747103
Mouse T-bet BV786 O4-46 1:100 BD 564141
Mouse EOMES PerCP-eFluor 710 Dan11mag 1:200 eBioscience 46-4875-82
Mouse TOX PE REA473/TXRX10 1:600 Miltenyi Biotech 130-120-716
Mouse TOX APC REA473/TXRX10 1:600 Miltenyi Biotech 130-118-335
Mouse CTLA4 PE-Dazzle594 UC10-4B9 1:400 BioLegend 106318
Mouse CD69 PE-Cy5 H1.2F3 1:1000 BioLegend 104510
Mouse TCF1 PE-Cy7 C63D9 1:600 Cell Signaling Technology 90511S
Mouse SLAMF6 APC 13G3-19D 1:200 eBioscience 17-1508-82
Mouse SLAMF6 PE 13G3 1:200 BD 561540
Mouse BCL-2 Alexa Fluor 647 BCL/10C4 1:200 BioLegend 633510
Mouse Granzyme B Alexa Fluor 700 QA16A02 1:200 BioLegend 372222
Mouse CX3CR1 APC-Fire 750 SA011F11 1:400 BioLegend 149040
Mouse CD39 PerCP-Cy5.5 Y23-1185 1:200 BD 567270
Mouse CD4 APC-Fire810 GK1.5 1:400 BioLegend 100480
Mouse IFN-γ PE-Cy7 XMG1.2 1:1000 BioLegend 505826
Mouse TNF-? APC MP6-XT22 1:2000 BioLegend 506308
Mouse Perforin FITC S16009A 1:100 BioLegend 154310
Mouse IL-2 PE A21001C 1:100 BioLegend 606553
Mouse CD101 PE-Cy7 S18006K 1:200 BioLegend 158210
Mouse CD127 PE A7R34 1:200 BioLegend 135010
Mouse Granzyme A PE 3G8.5 1:800 BioLegend 149703
Mouse 2B4 PE-Cy7 m2B4 (B6)458.1 1:200 BioLegend 133512
Mouse ZFP148 Alexa Fluor 647 H-7 1:400 Santa Cruz Biotechnology sc-137171
Mouse ZFP148 PE H-7 1:400 Santa Cruz Biotechnology sc-137171
Mouse KLF2 PE E7K8Y 1:200 Cell Signaling Technology 51221S
Mouse H-2D(b) LCMV gp33-41 Tetramer BV421 N/A 1:100 NIH Tetramer Core Facility N/A
Mouse H-2D(b) LCMV gp276-286 Tetramer PE N/A 1:100 NIH Tetramer Core Facility N/A

Human CD45 BV510 2D1 1:200 BioLegend 368526
Human CD3 BV570 UCHT1 1:100 BioLegend 300436
Human CD8 Super bright 436 OKT8 1:100 eBioscience 62-0086-42
Human CD4 APC Fire 810 SK3 1:400 BioLegend 344662
Human FOXP3 eFluor 450 PCH101 1:200 eBioscience 48-4776-41
Human CD11b BUV661 M1/70 1:5000 BD 612977
Human CD56 BV750 5.1H11 1:200 BioLegend 362556
Human CD45RA AF532 HI100 1:200 eBioscience 56-0458-42
Human CD45RO BB515 UCHL1 1:200 BD 564529
Human CD25 sup600 BC96 1:100 eBioscience 63-0259-42
Human PD-1 BUV737 EH12.1 1:100 BD 612791
Human TIM-3 BV711 7D3 1:800 BD 565567
Human TOX APC REA473/TXRX10 1:200 Miltenyi 130-118-335
Human TCF1 PE 7F11A10 1:200 BioLegend 655208
Human CD62L BV421 DREG-56 1:800 BD 563862
Human CTLA4 PEDAZZLE 594 BNI3 1:100 BioLegend 369616
Human LAG-3 PEcy5 3DS223H 1:100 eBioscience 15-2239-42
Human KLRG1 PE-Cy7 MAFA 1:800 BioLegend 138416
Human T-bet BV786 O4-46 1:100 BD 564141
Human Ki-67 BUV395 B56 1:200 BD 564071
Human Granzyme B AF700 N4TL33 1:100 eBioscience 58-8896-42
Human ICOS AF488 C398.4A 1:100 BioLegend 313514
Human CD69 BUV805 FN50 1:100 BD 748763
Human NKG2D BV480 1D11 1:100 BD 746404
Human NKG2A BUV615 131411 1:100 BD 752302
Human TIGIT BV650 741182 1:200 BD 747840
Human KIR2DL1 APCCy7 HP-MA4 1:200 BioLegend 339520
Human KIR3DL1 BUV563 DX9 1:100 BD 748923
Human CD27 SPARK NIR 685 O323 1:400 BioLegend 302856
Human CCR7 BUV496 2-L1-A 1:100 BD 749827
Human BCL2 AF647 Bcl-2/100 1:800 BD 563600
Human EOMES PE-Cy5.5 WD1928 1:400 eBioscience 35-4877-42
Human CD28 percp5.5 CD28.2 1:100 eBioscience 45-0289-42
Human ZFP148 Alexa Fluor 488 H-7 1:400 Santa Cruz Biotechnology sc-137171
Human IFN-γ FITC 4S.B3 1:400 BioLegend 502506
Human TNF-? PE-Cy7 MAb11 1:200 BioLegend 502930
Human IL-2 PE MQ1-17H12 1:200 eBioscience 12-7029-42

CUT&Tag-seq antibodies
Rabbit Anti-ZBP89 (ZFP148) Bethyl Laboratories # A303-116A
Anti-Rabbit Secondary Antibody EpiCypher, # SKU: 13-0047
Rabbit IgG Negative Control Antibody EpiCypher, # SKU: 13-0042

Cell culture antibodies
anti-mouse CD3ε 145-2C11 BioLegend
anti-mouse CD28 37.51 BioLegend

Validation

All antibodies were obtained from reputable and established commercial sources. Prior to experimental use, each antibody was tested and titrated to ensure optimal performance. Additional validation details are available on the manufacturers' websites using the catalog numbers provided in the supplementary materials. For flow cytometry, antibody specificity was validated using fluorescence minus one (FMO) controls and appropriate co-staining strategies.

Eukaryotic cell lines

Policy information about cell lines and Sex and Gender in Research

Cell line source(s)

B16-GP cell line was kindly provided by A. Wieland at The Ohio State University. B16-GP cells were cultured in RPMI 1640 medium (Gibco, 11875-093) with 10% heat-inactivated fetal bovine serum (FBS) (Gibco, 10082-147) and 1% penicillin/streptomycin (Gibco, 15140-122). The MC38 cell line was purchased from Kerafast (ENH204-FP). MC38 cells were cultured in Dulbecco's modified Eagle's medium (DMEM; Gibco, 11965-092) with 10% FBS and 1% penicillin/streptomycin. EL4 cell line was kindly provided by K. Oestreich at The Ohio State University. EL4 cells were cultured in RPMI-1640 with 10% FBS and 1% penicillin/streptomycin.

Authentication

MC38, B16-GP and EL4 cells were not independently authenticated

Mycoplasma contamination

All cell lines were tested negative for Mycoplasma contamination.

Commonly misidentified lines
(See ICLAC register)

N.A.

Animals and other research organisms

Policy information about studies involving animals; ARRIVE guidelines recommended for reporting animal research, and Sex and Gender in Research

Laboratory animals	C57BL/6J (strain 000664) mice were obtained from the Jackson Laboratory. CD8-specific ZFP148-deficient mice were generated by crossing E8iCre (the Jackson Laboratory, strain 008766) mice with Zfp148fl/fl mice kindly provided by J. L. Merchant at University of Arizona. P14 mice were kindly provided by S. M. Kaech at The Salk Institute for Biological Studies. KLF2GFP reporter mice were kindly provided by S. C. Jameson at the University of Minnesota via W. Cui at Northwestern University and crossed with P14 mice at Northwestern University to generate P14 KLF2-EGFP mice.
Wild animals	This study did not involve wild animals
Reporting on sex	Both male and female mice aged 8–10weeks were used.
Field-collected samples	No field collected samples were used in this study
Ethics oversight	Mice were maintained in the animal facility at The Ohio State University under standard conditions (ambient temperature 20–24°C, relative humidity 30–70%, 12-h dark–light cycle (lights on from 6:00 to 18:00)). Both male and female mice aged 8–10weeks were used. All procedures were performed in strict accordance with the NIH Guide for the Care and Use of Laboratory Animals and approved by the Committee on the Ethics of Animal Experiments of The Ohio State University. The Ohio State University Institutional Animal Care and Use Committee (IACUC; protocol 2019A00000075), Institutional Biosafety Committee (IBC; protocol 2019R00000046)

Note that full information on the approval of the study protocol must also be provided in the manuscript.

Plants

Seed stocks	*Report on the source of all seed stocks or other plant material used. If applicable, state the seed stock centre and catalogue number. If plant specimens were collected from the field, describe the collection location, date and sampling procedures.*
Novel plant genotypes	*Describe the methods by which all novel plant genotypes were produced. This includes those generated by transgenic approaches, gene editing, chemical/radiation-based mutagenesis and hybridization. For transgenic lines, describe the transformation method, the number of independent lines analyzed and the generation upon which experiments were performed. For gene-edited lines, describe the editor used, the endogenous sequence targeted for editing, the targeting guide RNA sequence (if applicable) and how the editor was applied.*
Authentication	*Describe any authentication procedures for each seed stock used or novel genotype generated. Describe any experiments used to assess the effect of a mutation and, where applicable, how potential secondary effects (e.g. second site T-DNA insertions, mosiacism, off-target gene editing) were examined.*

ChIP-seq

Data deposition

☒ Confirm that both raw and final processed data have been deposited in a public database such as GEO.

☒ Confirm that you have deposited or provided access to graph files (e.g. BED files) for the called peaks.

Data access links<br>*May remain private before publication.*	CUT&Tag-seq data are available in the NCBI database under accession numbers GSE296311 (https://www.ncbi.nlm.nih.gov/geo/query/acc.cgi?acc=GSE296311)
Files in database submission	ZFP148_R1_001.fastq.gz<br>ZFP148_R2_001.fastq.gz<br>IgG_R1_001.fastq.gz<br>IgG_R2_001.fastq.gz<br>ZFP148.bigWig<br>IgG.bigWig
Genome browser session<br>(e.g. UCSC)	https://tinyurl.com/2s43vcju

Methodology

Replicates	CUT&Tag-seq data are from single replicate. Each replicate was pooled together from 5 mice.
Sequencing depth	50 to 70 million reads were generated for each library.
Antibodies	Rabbit Anti-ZBP89 (ZFP148) Bethyl Laboratories # A303-116A<br>Anti-Rabbit Secondary Antibody EpiCypher, # SKU: 13-0047<br>Rabbit IgG Negative Control Antibody EpiCypher, # SKU: 13-0042

Peak calling parameters	Peaks were called using SEACR (v.1.3)
Data quality	All samples passed FastQC sequencing read quality assessment.<br>133483 peaks called for the ZFP148 sample
Software	CUT&Tag sequencing data were processed using the nf-core/cutandrun pipeline (v3.0.0) [https://nf-co.re/cutandrun/3.2.2/], a community-curated Nextflow pipeline. Raw sequencing reads were first subjected to adapter trimming using fastp (v0.23.2), followed by alignment to the mouse reference genome (mm10) using Bowtie2 (v2.4.4). Aligned reads were filtered to remove low-quality mappings, PCR duplicates (using Picard MarkDuplicates v2.27.5), and mitochondrial reads. Peaks were called using SEACR (v1.3) in "relaxed" mode with appropriate IgG or input control normalization. Genome-wide signal tracks were generated using deepTools (v3.5.1) and IGV (v2.18.4)for visualization. Quality control metrics, including fragment size distribution, duplication rates, and library complexity, were assessed and summarized using MultiQC (v1.13). All steps were run with default settings unless otherwise specified.

Flow Cytometry

Plots

Confirm that:

☐ The axis labels state the marker and fluorochrome used (e.g. CD4-FITC).

☒ The axis scales are clearly visible. Include numbers along axes only for bottom left plot of group (a 'group' is an analysis of identical markers).

☒ All plots are contour plots with outliers or pseudocolor plots.

☒ A numerical value for number of cells or percentage (with statistics) is provided.

Methodology

Sample preparation	Mouse samples<br>Mouse spleens were mechanically disrupted, washed once with ice-cold PBS, subjected to red blood cell lysis (BioLegend, 420302), passed through 70-µm cell strainers, and resuspended as single-cell suspensions.<br>For liver lymphocyte isolation, mice were perfused with ice-cold PBS via the hepatic portal vein. Livers were mechanically dissociated through 100-µm strainers, followed by lymphocyte isolation using Percoll gradient centrifugation (44% Percoll in RPMI over 67% Percoll in PBS; 450 × g for 20 min at room temperature) and red blood cell lysis.<br>For lung lymphocyte isolation, mice were perfused with ice-cold PBS. Lungs were minced and digested with Collagenase Type III (Worthington Biochemical Corporation, LS004182) for 90 min at 37 °C, passed through 70-µm strainers, and lymphocytes were isolated by Percoll gradient centrifugation (44% Percoll in RPMI over 67% Percoll in PBS; 500 × g for 20 min at room temperature).<br>Tumors were mechanically dissociated and digested with Collagenase Type I and Collagenase Type IV (Worthington Biochemical Corporation, LS004196 and LS004188) for 30 min at 37 °C with shaking at 125 rpm. Digestion was quenched with ice-cold PBS containing 2% bovine serum albumin, and red blood cell lysis (BioLegend) was performed as needed before filtration through 70-µm strainers.<br><br>Human samples<br>Clinical specimens were processed on the day of radical cystectomy. Tumor tissue was manually dissociated, centrifuged (600 × g for 5 min at 4°C), resuspended in human tumor dissociation enzyme solution (Miltenyi Biotec, 130-095-929), and homogenized using a gentleMACS semi-automated dissociator (Miltenyi Biotec). Homogenized tissue was incubated at 37°C for 20 min under continuous rotation on a MACSmix Tube Rotator (Miltenyi Biotec). Following addition of 2% bovine serum albumin (BSA), single-cell suspensions were filtered twice through a 70 µm cell strainer, washed with PBS, subjected to red blood cell lysis (BioLegend, 420302), and resuspended in RPMI medium.<br>Peripheral blood was collected in heparin–EDTA tubes and processed by Ficoll–Hypaque (Sigma) density gradient centrifugation to isolate peripheral blood mononuclear cells (PBMCs).
Instrument	Cytek® Aurora, Cytek Aurora™ CS System
Software	All results were analyzed with FlowJo software (v10.7.1, TreeStar) or OMIQ Flow Cytometry software (Dotmatics).
Cell population abundance	For sorting with Cytek Aurora™ CS System, cells were reanalyzed after sorting, achieving and confirming a purity of >90%.
Gating strategy	The gating strategy for sorting out CD44hiGP33–41 Tet+CD8+ T cells from spleens of Zfp148fl/fl and ZFP148 cKO mice infected with LCMV Cl13 is shown in Extended Data Fig. 6a.<br>For in vitro–stimulated mouse or human CD8+ T cells, lymphocytes were first identified using SSC-A versus FSC-A based on cell size and granularity. Doublets were excluded by FSC-H versus FSC-A gating. Live CD8+ T cells were then selected by excluding viability dye–positive cells and gating on CD8+ cells.<br>For CD44hiGP33–41 Tet+CD8+ T cells or CD44hiGP276–286 Tet+CD8+ T cells from spleens or inguinal lymph nodes of C57BL/6 WT, Zfp148fl/fl, or ZFP148 cKO mice following LCMV Cl13 or LCMV Armstrong infection, lymphocytes were first identified by SSC-A versus FSC-A, and doublets were excluded as described above. Live CD45+ immune cells were then selected, followed by CD3+ T cells (CD3+CD11b−), CD8+ T cells (CD8+CD4−), and finally CD44hi tetramer-positive antigen-specific populations.<br>For analysis of transferred P14 or P14 KLF2-EGFP CD8+ T cells in spleens of C57BL/6 WT recipient mice at day 21 post-transfer and LCMV Cl13 infection, lymphocytes were first identified by SSC-A versus FSC-A, and doublets were excluded as above. Live CD45+ immune cells were selected, followed by CD3+ T cells (CD3+CD11b−), CD8+ T cells (CD8+CD4−), and

transferred CD45.1+ P14 or P14 KLF2-EGFP cells.
For mouse tumor-infiltrating CD8+ T cells, lymphocytes were first identified by SSC-A versus FSC-A, and doublets were excluded as above. Live CD45+ immune cells were selected, followed by CD3+ T cells (CD3+CD11b−) and CD8+ T cells (CD8 +CD4−).
For human CD8+ T cells isolated from peripheral blood mononuclear cells or muscle-invasive bladder tumors, lymphocytes and singlets were gated as described above. Live CD45+ immune cells were selected, followed by CD3+ T cells (CD3+CD11B−) and CD8+ T cells (CD8+CD4−). Activated or naïve CD8+ T cells were defined as CD45RA−CD45RO+ or CD45RA+CD45RO− populations, respectively.
Appropriate fluorescence minus one (FMO) controls and positive controls were included in each experiment to define gating boundaries.

☒ Tick this box to confirm that a figure exemplifying the gating strategy is provided in the Supplementary Information.
